# Supplementary material for: Evolution, geographic spreading, and demographic distribution of Enterovirus D68
Source: PLoS Pathog. 2022 May 31;18(5):e1010515. doi: 10.1371/journal.ppat.1010515 (PMC9212145; doi:10.1371/journal.ppat.1010515)
Supplement: S1 Text — Detailed descriptions of the patient populations and sampling methods, datasets, sequencing, bioinformatics, and phylogenetic analysis, and the analyses of age, diversity, and geographic spread. (PDF) [file ppat.1010515.s001.pdf]

Evolution, geographic spreading, and demographic distribution of Enterovirus D68: Supplementary Text

Emma B. Hodcroft,[1,2,3,*] Robert Dyrdak,[4,5] Cristina Andrés,[6] Adrian Egli,[7,8] Josiane Reist,[7,8] Diego García Martínez de Artola,[9] Julia Alcoba-Flórez,[9] Hubert G. M. Niesters,[10] Andrés Antón,[6] Randy Poelman,[10] Marijke Reynders,[11] Elke Wollants,[12] Richard A. Neher,[1,3] and Jan Albert[4,5]

[1] *Biozentrum, University of Basel, Basel, Switzerland*

[2] *Institute of Social and Preventive Medicine, University of Bern, Bern, Switzerland*

[3] *Swiss Institute of Bioinformatics, Basel, Switzerland*

[4] *Department of Clinical Microbiology, Karolinska University Hospital, Stockholm, Sweden*

[5] *Department of Microbiology, Tumor and Cell Biology, Karolinska Institute, Stockholm, Sweden*

[6] *Respiratory Viruses Unit, Virology Section, Microbiology Department, Vall d'Hebron Hospital Universitari, Vall d'Hebron Institut de Recerca (VHIR), Vall d'Hebron Barcelona Hospital Campus, Barcelona, Spain*

[7] *Clinical Bacteriology and Mycology, University Hospital Basel, Basel, Switzerland*

[8] *Applied Microbiology Research, Department of Biomedicine, University of Basel, Basel, Switzerland*

[9] *Department of Clinical Microbiology, Hospital Universitario Nuestra Señora de Candelaria, Tenerife, Spain*

[10] *University of Groningen, University Medical Center Groningen, Department of Medical Microbiology, Division of Clinical Virology, Groningen, The Netherlands*

[11] *Unit of Molecular Microbiology, Medical Microbiology, Department of Laboratory Medicine, AZ Sint-Jan Brugge AV, Bruges, Belgium*

[12] *KU Leuven, Rega Institute, Department of Microbiology, Immunology and Transplantation, Laboratory of Clinical & Epidemiological Virology, Leuven, Belgium*

* Correspondence to: Dr. Emma Hodcroft, ISPM, Mittelstrasse 43, 3012, Bern, Switzerland. **emma.hodcroft@ispm.unibe.ch**

I. SUPPLEMENTARY MATERIALS AND METHODS

A. Patient population/samples.

Respiratory samples positive for EV-D68 were collected from six virology laboratories (see Table 1). Ethical approvals and consents were obtained as required by local regulations (see below).

In Stockholm, EV-D68 testing was done on all respiratory samples positive for enterovirus from 1 August to 31 October 2018 at the Karolinska University Laboratory, which serves 6 of 8 emergency hospitals in the Stockholm county. An additional 25 enterovirus-positive samples from the pediatric ICU at Karolinska University Hospital collected during November 2018 were also tested. Specific EV-D68 testing was done using a published real-time PCR [1]. All samples positive with an in-house EV-D68-specific qPCR [1] were included. Eluates were obtained from extraction on an automated system (MagNA Pure [Roche]). RNA extraction of samples shipped to Stockholm from the other study centers was done by manually using the RNeasy Lipid Tissue Mini Kit (Qiagen cat. No. 74804). The study was reviewed and approved by the Regional Ethical Review Board in Stockholm, Sweden (registration no. 2017/1317–32). No consent was required for samples, including from children, as they are anonymous.

In Groningen, testing was done routinely (year around) using a specific assay for rhinovirus or enterovirus, or a combination of rhinovirus/enterovirus (FilmArray RP2, BioFire). If positive, a specific enterovirus D68 assay was used. A selection of 10 samples with low Ct-values of a total of 21 positive samples in 2018 were sent to Stockholm (Ethical Approval METc 2017/278). No consent was required for samples, including from children, as they are anonymous.

In Belgium, for the first time, there was an upsurge of EV-D68 in 2018 [2]. At Leuven, 10 positive EV-D68 samples with low Ct-values, sent from AZ Sint. Jan Brugge, were confirmed with a nested VP4/VP2 PCR [3]. This hospital in Bruges detected 83 positive EV-D68 samples in 7,986 respiratory samples in 2018 by using TAC (Taqman Array Card) technology for broad respiratory screening. In 2015 a specific real-time PCR for EVD-68 was integrated on this microarray card [4]. In the context of a national reference laboratory for enteroviruses, no ethical approval was needed, including for samples from children.

In Basel, 157 samples were screened using an in-house PCR based on primers by Piralla *et al.* [5]. The six samples sent to sent to Stockholm represent all positive samples. The catchment area of the laboratory did not include pediatric departments. For University Hospital Basel samples, only anonymized samples without additional patient data were used, which does not require a specific ethical evaluation in accordance with correspondence with Swissethics. No consent was required for samples, including from children, as they are anonymous.

In Barcelona, detection of EV in respiratory specimens was performed by specific real-time multiplex RT-PCR assay (Allplex Respiratory Panel Assay, Seegene, Korea). EV were characterized by phylogenetic analyses based on VP1 sequence, as previously described [6]. A selection of 14 positive samples with low Ct-values of a total of 44 EV-D68 positive specimens from 2018 were used in this study. Institutional Review Board approval (PR(AG)173/2017) was obtained from the HUVH Clinical Research Ethics Committee. No consent was required for samples, including from children, as they are anonymous.

In Tenerife, primary detection of enterovirus was done by specific real-time multiplex RT-PCR assay (Allplex Respiratory Panel Assay, Seegene, Korea) with subsequent typing of positive samples by VP1 sequencing [7]. Consent for further analysis was obtained for nine of a total of twelve positive samples. Eluates of the nine samples, obtained from extraction on an automated system (EasyMag-EMag [Biomerieux]), were sent to Stockholm. Local Research Ethics Committee approval was obtained (code CHUNSC_2019_02). Consent was obtained verbally from parents/guardians for samples from children.

B. Sequencing and bioinformatic processing

Near full-length genome sequencing ('whole genome' sequencing) was performed as previously described [8]. Briefly, the genome was amplified in duplicate by one-step RT-PCR in four overlapping fragments. Duplicates of each fragment were pooled and purified using AGENCOURT AMPure XP PCR purification kit and quantified with Qubit assays (Q32851, Life Technologies). Purified DNA from each fragment was diluted to the same concentration, pooled and sent to the Clinical Genomics Unit at Science for Life Laboratory for library preparation and sequencing (SciLifeLab, Stockholm, Sweden).

In total 1 μl of DNA ($\sim$0.5–2.0 ng/μl) was used in the tagmentation reaction using

Nextera chemistry (Illumina) to yield fragments >150 bp. The tagmented library underwent eleven cycles of PCR with single-end indexed primers (IDT Technologies) followed by purification using Seramag beads. The library was quantified using Quant-iT dsDNA High-Sensitivity Assay Kit and Tecan Spark 10 M or FLUOstar Omega plate reader. The library was then pair-end sequenced to a depth of 100,000–1,500,000 reads per sample on either HiSeq 2500 ($2 \times$ 101bp) or NovaSeq 6000 ($2 \times$ 151bp) Illumina sequencers. Base calling and demultiplexing was done using bcl2fastq v1.87, without allowing any mismatch in the index sequence. Assembly was done as described previously [8], but to improve mapping sensitivity, we replaced BWA by NextGenMap [9] and used mapping references from the same subclade as the sample. The scripts implementing this workflow are available on github at github.com/neherlab/EV-D68_sequence_mapping. Fifty-two of 55 samples were sequenced with a coverage of $>100\times$ in all four fragments and were included in the further analysis along with one sample with a coverage of $>10\times$ in one fragment and $>100\times$ in the other three fragments, giving a total of 53 successfully sequenced samples. The consensus sequences for these 53 samples have been deposited in GenBank (accession numbers MN245396-MN245448). The raw reads have been deposited in the Short Read Archive (BioProject number PRJNA525063, BioSample accession numbers SAMN13745166-SAMN13745216). A list of accession numbers, along with metadata is available as S1 Table.

C. Whole Genome and VP1 Sequence Data Sets

The consensus sequences from the 53 samples sequenced in this study were combined with whole genomes with length >6000bp available in the Virus Pathogen Resource (ViPR) [10] (as of 2019-09-12) as well as samples matching this criterion manually curated from GenBank. Of all the sequences available in GenBank (n=4,259, on 1 Nov 2019), 70% were annotated with an isolation source, of which the majority were respiratory specimens (88%).

To conduct additional analyses on an as large and representative a data set as possible, a further data set of VP1 sequences was assembled. All EV-D68 sequences in ViPR were downloaded and BLASTed [11] against a 927 bp reference VP1 alignment (KX675261). Only matching regions of at least 700 bp and an Expect Value (E-value) of 0.005 or lower were included. All sequences from the whole genome data set were included in the VP1 dataset.

To counter over-representation of countries with high sample numbers during some time

periods, the VP1 dataset was down-sampled using the `augur filter` command, randomly selecting at most 20 samples per month, per year, per country.

Though the 3 whole genome and 7 VP1 sequences sampled prior to 1990 fit the estimated molecular clock well when included in the analyses, they were omitted for figure clarity. Samples without a year of sampling were also excluded (3 from the whole genome run; 6 from the VP1 run), as well as mouse-adapted samples and extreme outliers (6 from the whole genome run; 31 from the VP1 run). The default settings in `augur tree` (IQTree, with a GTR substitution model) were used to generate initial phylogenies. `augur refine` was then used to generate time-resolved trees, branches more then 5 interquartile distances from the substitution rate regression were pruned, removing 3 and 4 sequences from the whole genome and VP1 datasets. The final number of sequences included the whole genome and VP1 phylogenies was 813 and 1,654, respectively. Accession numbers and author/publication details for each sequence included in the analyses are available at nextstrain.org/enterovirus/d68/genome and nextstrain.org/enterovirus/d68/vp1. A list of accession numbers, along with metadata is available as S3 and S4 Tables.

D. Age Data

In order to analyze associations between and patient age and EV-D68 clade and subclade, roughly 500 ages or age-ranges were manually scraped from over 40 papers. Over 100 additional ages were provided by authors to whom we reached out. Combined with age data available on GenBank, this resulted in approximately 900 VP1 sequences and over 450 whole genome sequences with some kind of age information.

As some age information was available only as an age-range, age data was automatically parsed to create an age range variable. 'Age' contains the exact decimal year, where available, and 'age range 1' consists of four categories (<1yr, 1-5yrs, 6-17yrs, 18-64yrs, and >=65yrs). For the borderline cases of age ranges given as '0-1' and '0-18', these were interpreted as <1yr and <18yrs, respectively. Data was available for exact age and 'age range 1' for 778 and 792 VP1 samples and 378 and 378 whole genome samples.

The effect of subclade designation on age was examined using the `lm` function in R [12] to perform simple linear models, with and without sample year and region as co-factors.

E. Phylogenetic analysis & Evolution

We used the augur pipeline [13] to analyze the whole genome and VP1 data sets. Briefly, sequences were aligned using mafft [14] and annotated according to the 1962 Fermon strain (GenBank accession AY426531), a phylogenetic tree was inferred using IQ-TREE [15], and maximum likelihood time trees were inferred using TreeTime [16]. Samples deviating from the estimated clock rate by more than 5 inter-quartile distances were removed during this step. Classification into clades and subclades was automated using the augur 'clades' command, based on mutations which matched the typing assigned to sequences by the Enterovirus Genotyping Tool 0.1 at `https://www.rivm.nl/mpf/typingtool/enterovirus/` [17] (see S5 and S6 Tables for mutations used to classify). The scripts implementing this workflow are available on github at github.com/nextstrain/enterovirus_d68. The scripts to produce the further analyses and figures for this paper are available at github.com/neherlab/2018_evd68_paneurope_analysis. Additionally, a frozen version of the phylogenies used in this manuscript can be viewed for the full-genome and VP1-gene in this repository.

The difference in number of mutations observed in the protein sequence of VP1-VP4 between exposed and buried or interior residues was calculated by Fisher exact test both for the polyprotein as a whole, and for the genes individually. Exposed residues on the outside of the viral capsid where determined by manual inspection of the biological assembly in PyMol and augmented by the un-modelled variable loops of VP1.

F. Diversification, Persistence, and Migration

To calculate the number of lineages leading to the samples taken during each season, a season-specific tree was created from the time-resolved, whole genome phylogeny, which contained all tips sampled during the outbreak year under investigation. Trees were assumed to be ultrametric, so the number of lineages at the end of the season is equal to the number of samples taken that year. Working backwards from the most recent sample, each coalescence event and the time is occurred was recorded, until only one lineage remained. To help show the similarity in lineage change between the seasons, they are plotted as years prior to the end of the outbreak year in Fig 2B.

Maximum likelihood estimates of migration rates were performed with TreeTime v0.7.0 using the command-line interface as explicitly documented in the Snakefile. In order to minimize bias by countries, regions, and years with very few samples, we estimated migration rates from VP1 trees pruned to only include tips from the outbreak year (2014, 2016, or 2018) under investigation, and metadata was masked to include only countries (in Europe) and regions with a relatively similar number of samples across outbreaks. For between-country migration estimates the countries were thus France, Spain, Germany, Italy, Sweden, “rest of Europe”, and “rest of world”, and for regions China, Europe, North America, and “rest of world.” However, estimates did not differ greatly from when all tips, countries, and regions were used. Similarly, estimates of the coalescent rate through time (aka “skyline”) was performed with TreeTime using $n = 150$ bins and restricting to full genome sequences after Jan 2011.

To color the VP1 tree by epitope patterns, variable amino acids at the specified locations were concatenated, and only those appearing more than 7 times (6 times for the C-terminus) were displayed. For the BC-loop these were positions 90, 92, 95, 97, 98, and 103; for the DE-loop these were positions 140-148. The C-terminus was not included in a large fraction of sequences resulting in many undetermined amino acids. Tips with more than 7 missing amino acid positions were grouped into a ‘many X’ category; missing amino acids were inferred from the parental sequence for patterns with 6 or fewer missing sites. The positions used were 280, 283, 284, 288, 290, 297, 299, 301, 304-306, and 308. The crystal structure in Fig 4 was generated using PyMOL [18]. Cumulative amino-acid changes are displayed along the branches of the colored trees in Fig S6 and Fig S8; these start at the earliest sequence for which a reliable amino-acid sequence is known (marked as ‘0’), and cumulatively count amino-acid changes (including reversions) along the branches.

REFERENCES

---

[1] Dyrdak R, Grabbe M, Hammas B, Ekwall J, Hansson KE, Luthander J, et al. Outbreak of enterovirus D68 of the new B3 lineage in Stockholm, Sweden, August to September 2016. Eurosurveillance. 2016;21(46).

[2] Wollants E, Beller L, Beuselinck K, Bloemen M, Lagrou K, Reynders M, et al. A decade of enterovirus genetic diversity in Belgium. J Clin Virol. 2019;121:104205.

[3] Wisdom A, Leitch EC, Gaunt E, Harvala H, Simmonds P. Screening respiratory samples for detection of human rhinoviruses (HRVs) and enteroviruses: comprehensive VP4-VP2 typing reveals high incidence and genetic diversity of HRV species C. J Clin Microbiol. 2009;47(12):3958–3967.

[4] Poelman R, Schölvinck EH, Borger R, Niesters HG, van Leer-Buter C. The emergence of enterovirus D68 in a Dutch University Medical Center and the necessity for routinely screening for respiratory viruses. Journal of Clinical Virology. 2015;62:1–5.

[5] Piralla A, Girello A, Grignani M, Gozalo-Margüello M, Marchi A, Marseglia G, et al. Phylogenetic characterization of enterovirus 68 strains in patients with respiratory syndromes in Italy. Journal of medical virology. 2014;86(9):1590–1593.

[6] Andres C, Vila J, Gimferrer L, Pinana M, Esperalba J, Codina MG, et al. Surveillance of enteroviruses from paediatric patients attended at a tertiary hospital in Catalonia from 2014 to 2017. J Clin Virol. 2019;110:29–35.

[7] Nix WA, Oberste MS, Pallansch MA. Sensitive, seminested PCR amplification of VP1 sequences for direct identification of all enterovirus serotypes from original clinical specimens. J Clin Microbiol. 2006;44(8):2698–2704.

[8] Dyrdak R, Mastafa M, Hodcroft EB, Neher RA, Albert J. Intra- and interpatient evolution of enterovirus D68 analyzed by whole-genome deep sequencing. Virus Evol. 2019;5(1):vez007.

[9] Sedlazeck FJ, Rescheneder P, von Haeseler A. NextGenMap: fast and accurate read mapping in highly polymorphic genomes. Bioinformatics. 2013;29(21):2790–2791.

[10] Pickett BE, Sadat EL, Zhang Y, Noronha JM, Squires RB, Hunt V, et al. ViPR: an open bioinformatics database and analysis resource for virology research. Nucleic acids research.

2011;40(D1):D593–D598.

[11] Altschul SF, Gish W, Miller W, Myers EW, Lipman DJ. Basic local alignment search tool. Journal of Molecular Biology. 1990;215(3):403–410. doi:10.1016/S0022-2836(05)80360-2.

[12] R Core Team. R: A Language and Environment for Statistical Computing; 2014. Available from: http://www.R-project.org/.

[13] Hadfield J, Megill C, Bell SM, Huddleston J, Potter B, Callender C, et al. Nextstrain: real-time tracking of pathogen evolution. Bioinformatics. 2018;doi:10.1093/bioinformatics/bty407.

[14] Katoh K, Misawa K, Kuma Ki, Miyata T. MAFFT: a novel method for rapid multiple sequence alignment based on fast Fourier transform. Nucleic Acids Research. 2002;30(14):3059–3066. doi:10.1093/nar/gkf436.

[15] Nguyen LT, Schmidt HA, von Haeseler A, Minh BQ. IQ-TREE: A Fast and Effective Stochastic Algorithm for Estimating Maximum-Likelihood Phylogenies. Molecular Biology and Evolution. 2015;32(1):268–274. doi:10.1093/molbev/msu300.

[16] Sagulenko P, Puller V, Neher RA. TreeTime: Maximum-likelihood phylodynamic analysis. Virus Evolution. 2018;4(1). doi:10.1093/ve/vex042.

[17] Kroneman A, Vennema H, Deforche K, Avoort H, Penaranda S, Oberste M, et al. An automated genotyping tool for enteroviruses and noroviruses. Journal of Clinical Virology. 2011;51(2):121–125.

[18] Schrödinger, LLC. The PyMOL Molecular Graphics System, Version 2; 2015.
