# Supplementary figures and images for: Evolution, geographic spreading, and demographic distribution of Enterovirus D68
Source: PLoS Pathog. 2022 May 31;18(5):e1010515. doi: 10.1371/journal.ppat.1010515 (PMC9212145; doi:10.1371/journal.ppat.1010515)

## Sample Date of Sequenced Samples in 2018

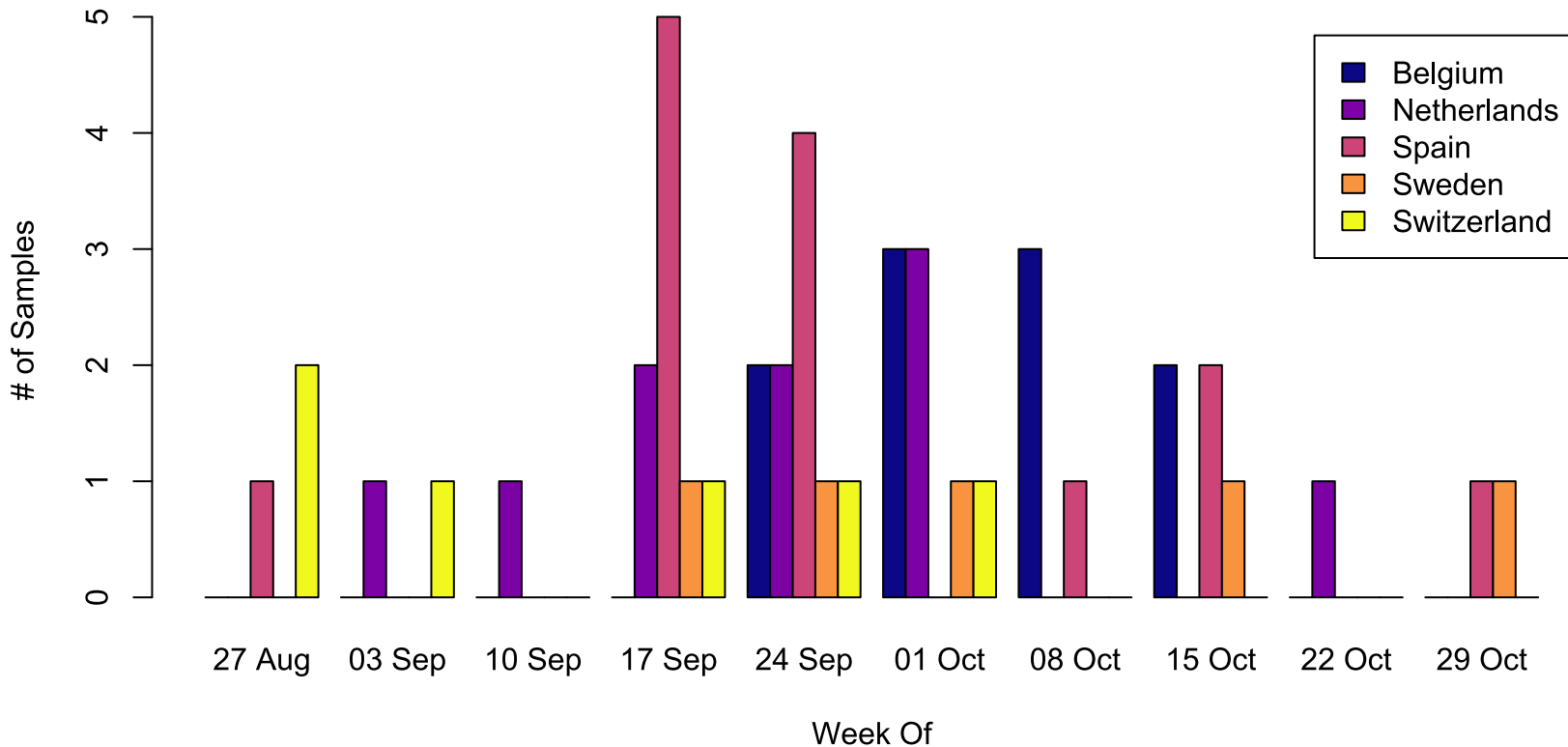

Supplement: S1 Fig — (PDF) [file ppat.1010515.s002.pdf]

## Age Distribution by Clade of 100 Bootstraps

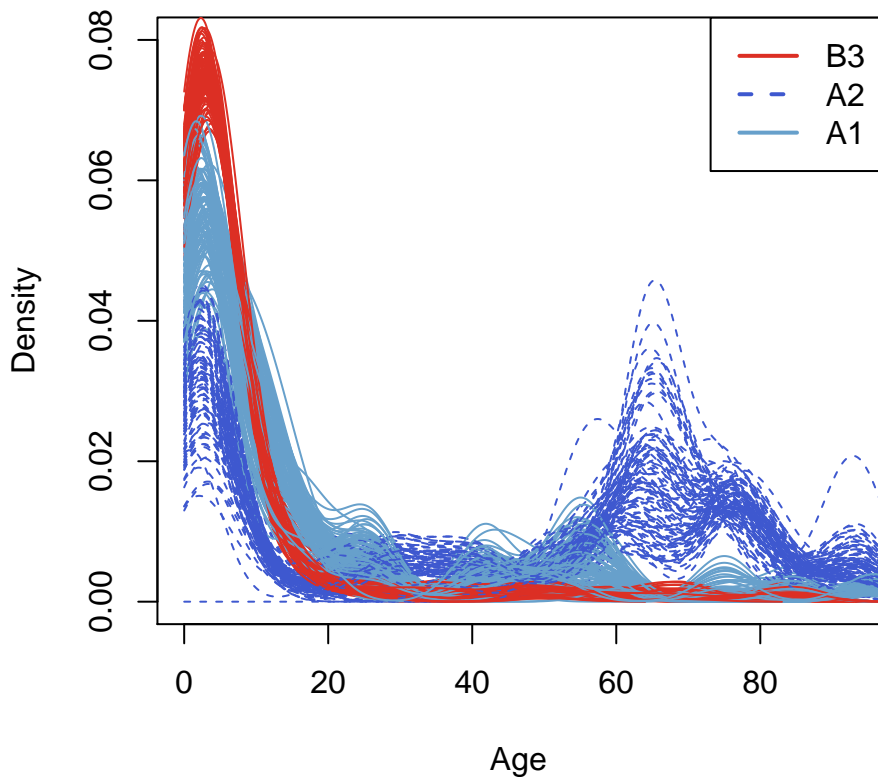

Supplement: S2 Fig — For each bootstrapped dataset, the same linear regression shown in Table 2 was performed, and the age distributions for the A1, A2, and B3 clades was plotted. The ages of the A2 clade was significantly different from the B3 clade in all bootstrap replicate linear regressions after Bonferroni correction. (PDF) [file ppat.1010515.s003.pdf]

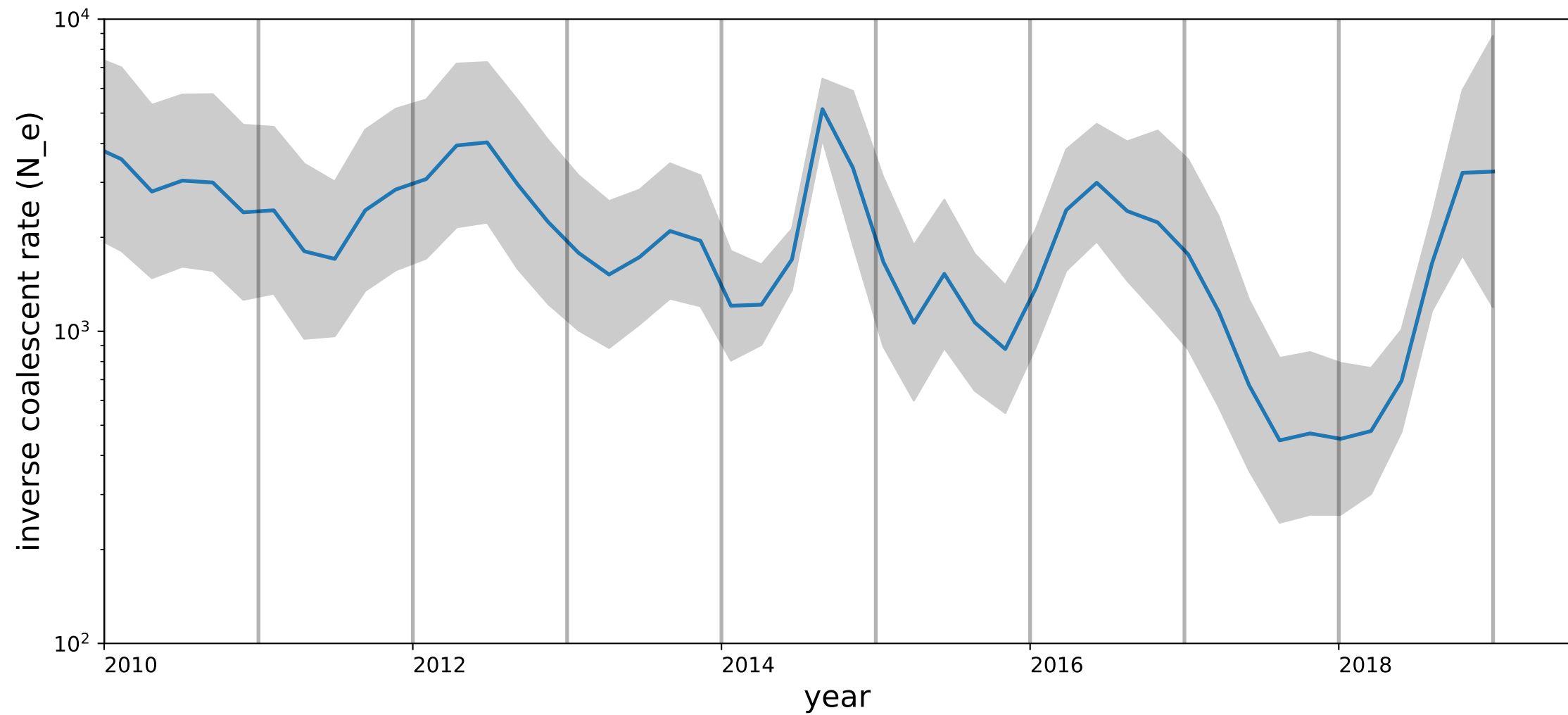

Supplement: S3 Fig — (PDF) [file ppat.1010515.s004.pdf]

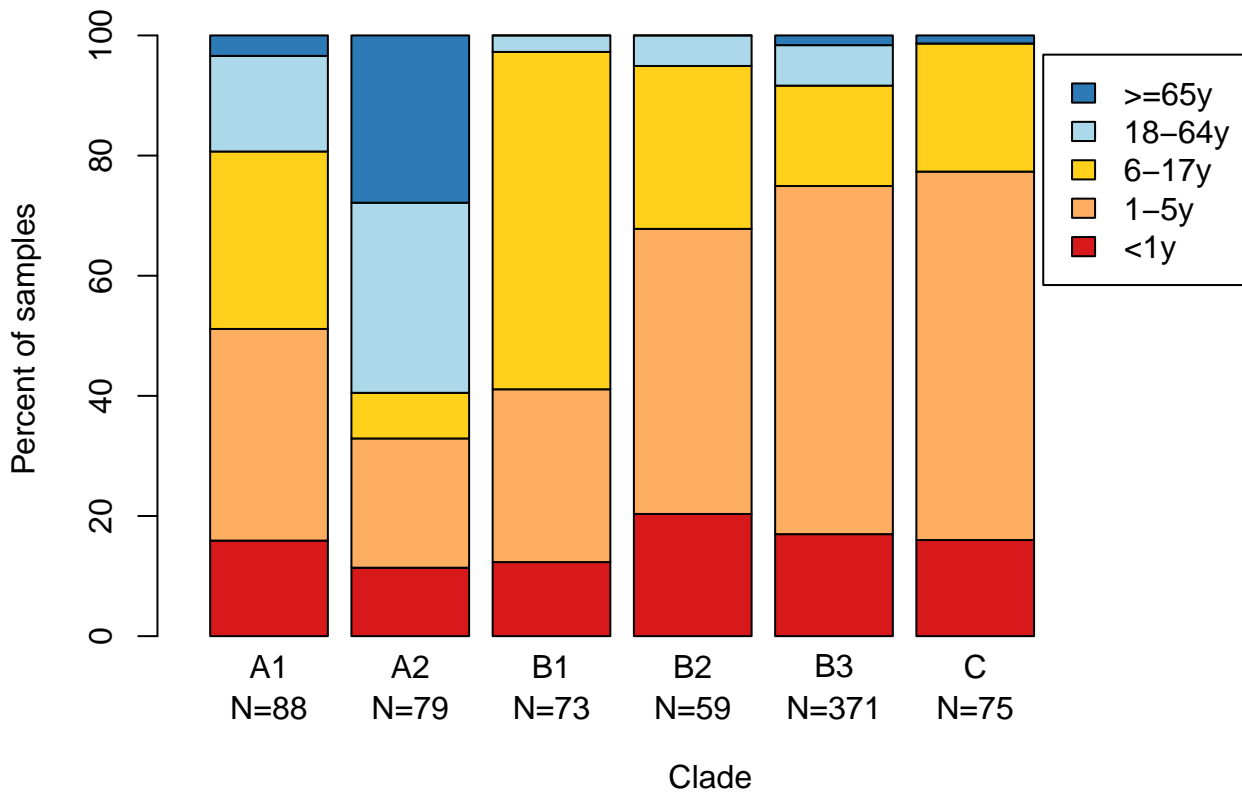

Supplement: S4 Fig — The over-representation of adults and the elderly in the A2 subclade can be seen clearly, along with the over-representation of adults (and to a lesser extent, the elderly) in the A1 subclade. (PDF) [file ppat.1010515.s005.pdf]

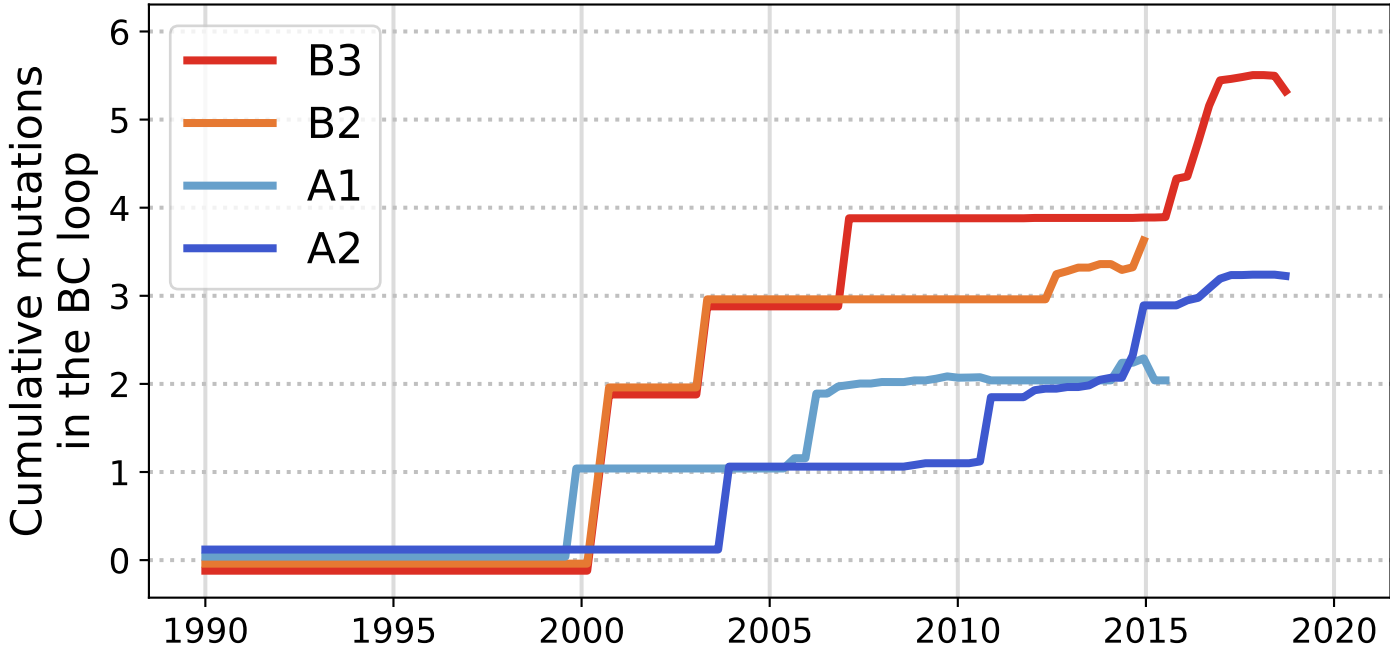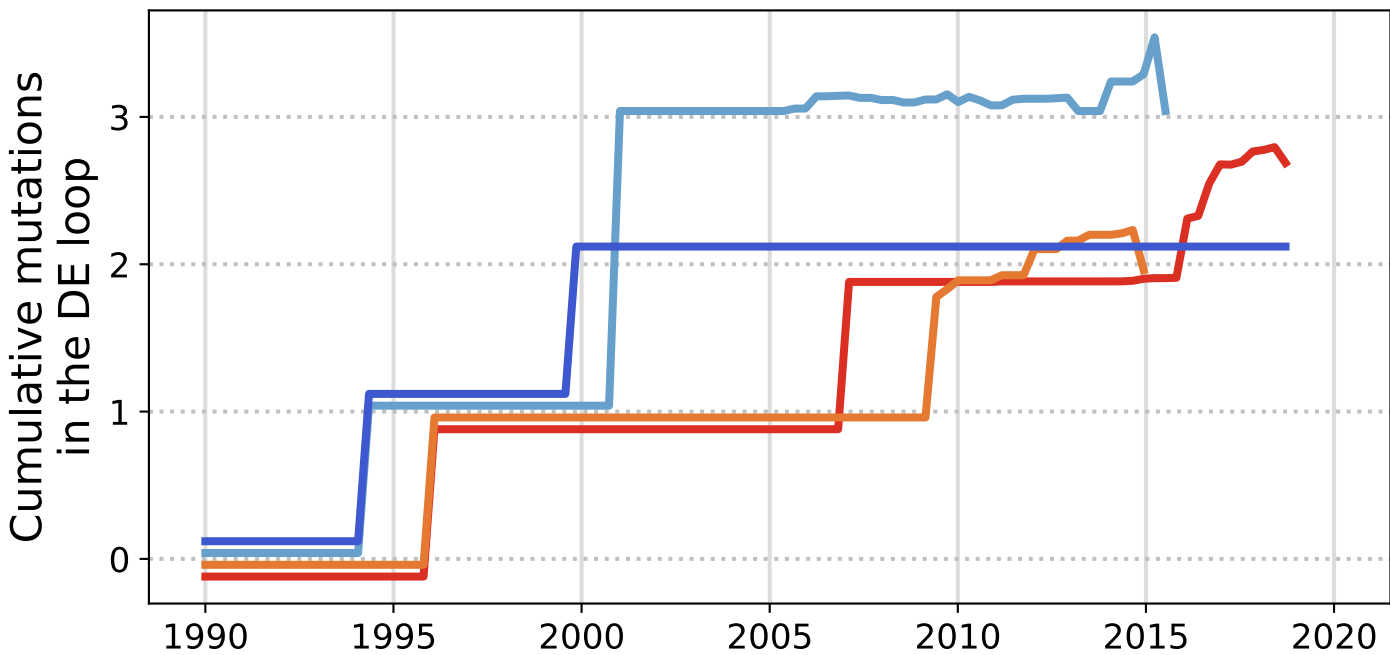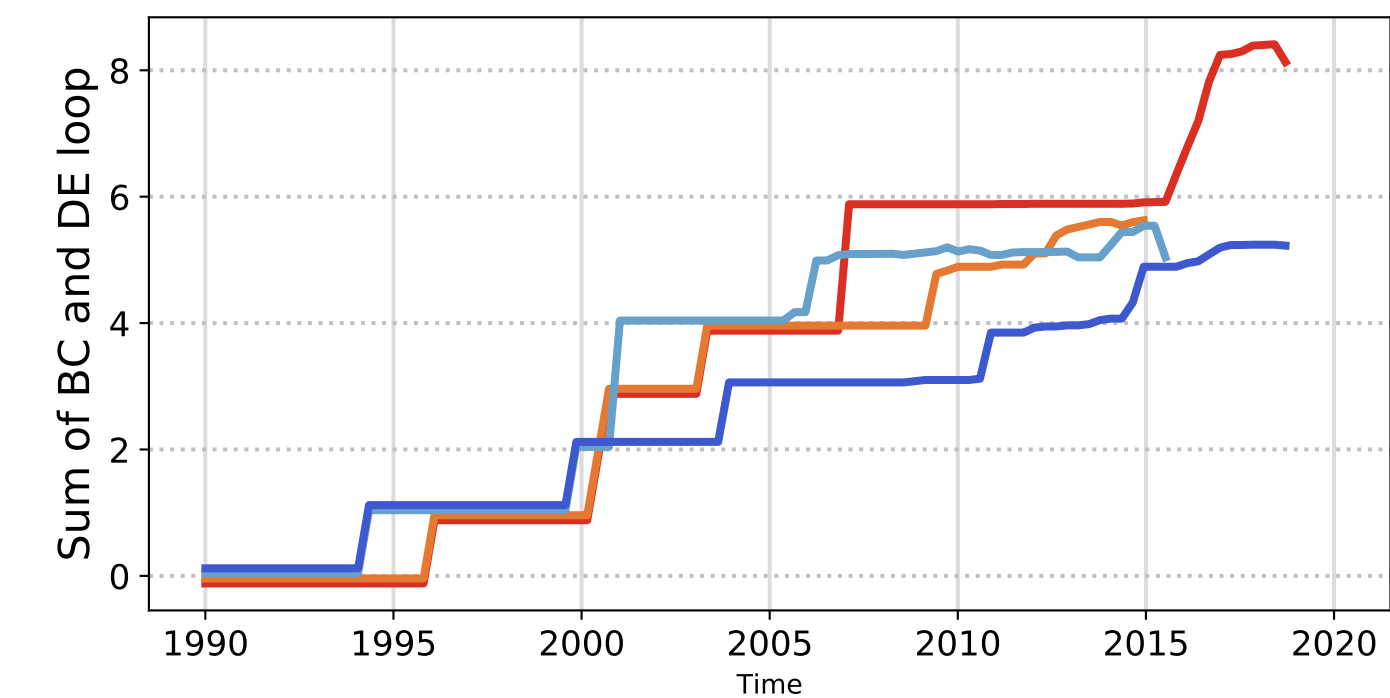

Supplement: S5 Fig — In the BC loop, AA positions 90, 92, 95, 97, 98, and 103, were used, and in the DE loop, AA positions 140–146 and 149 were used. The BC-loop plot (top) shows that the B3 clade had around 4 mutations between 2000 and 2008, then about 7 years without mutations. In contrast, the A2 clade had only one mutation prior to 2010, but then had two between 2011 and 2015. Mean mutation count lines have been plotted slightly above and below their true value so that all lines can be seen when they share the same value. Calculated for the whole genome dataset. (PDF) [file ppat.1010515.s006.pdf]

# Epitope At C Terminus ^

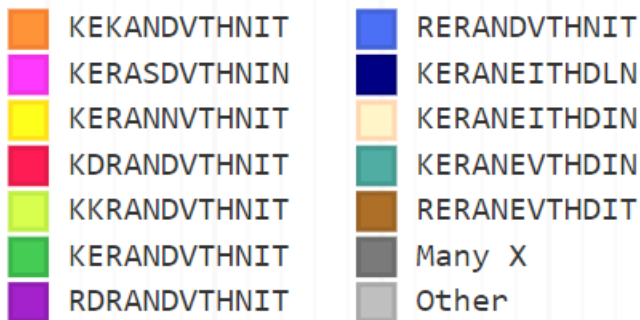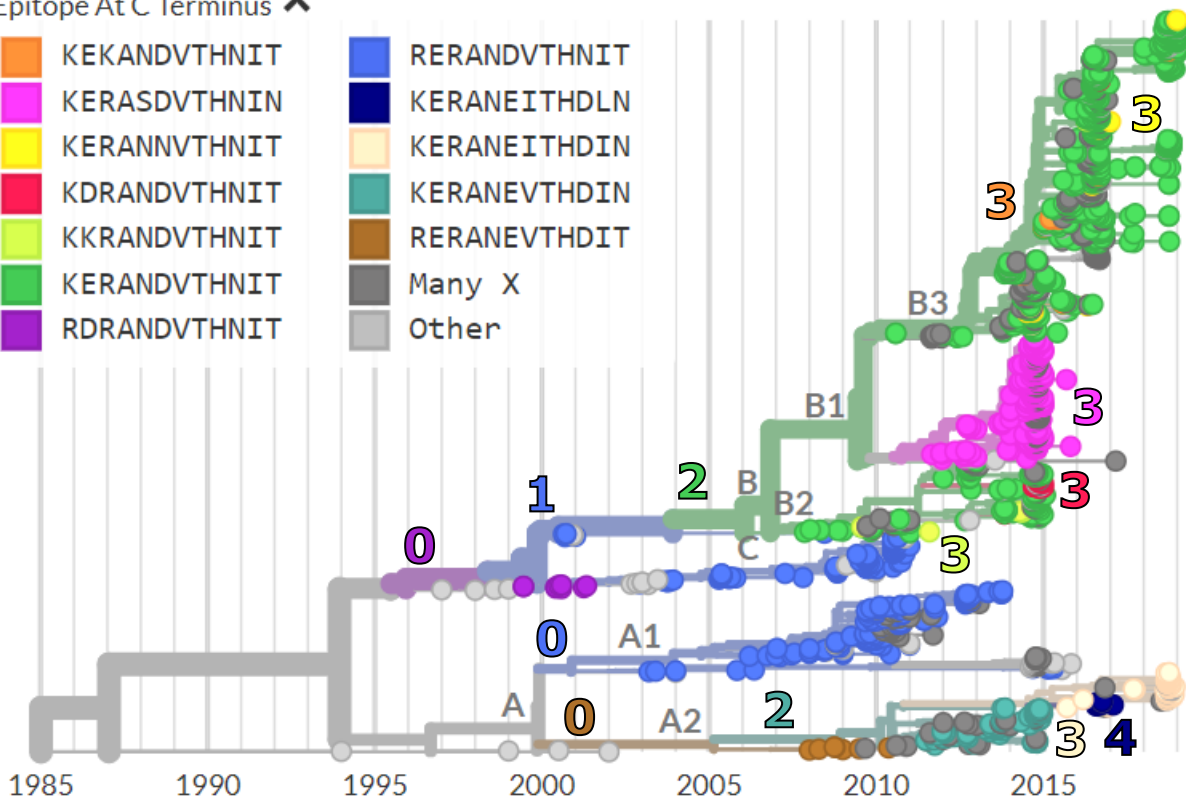

Supplement: S6 Fig — This figure extends from Fig 4D and 4E, but shows the most common C-terminus patterns at variable positions 280, 283, 284, 288, 290, 297, 299, 301, 304–306, and 308. Cumulative counts of the AA changes from the most recent reliably identifiable sequence (marked with ‘0’) are shown along the branches. As in the BC-loop in Fig 4, the A2 subclade shows substantial recent evolution in this region. (PDF) [file ppat.1010515.s007.pdf]

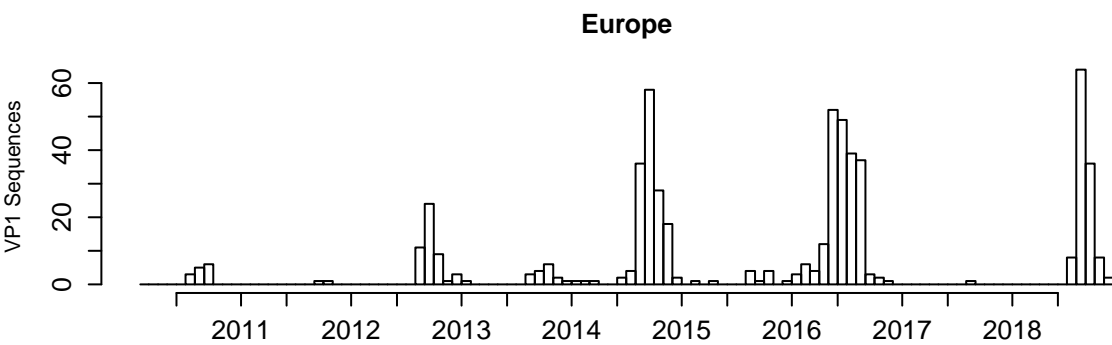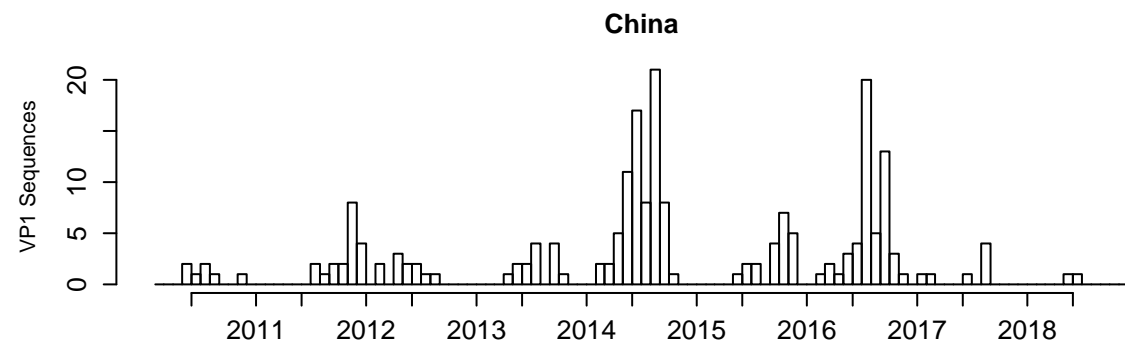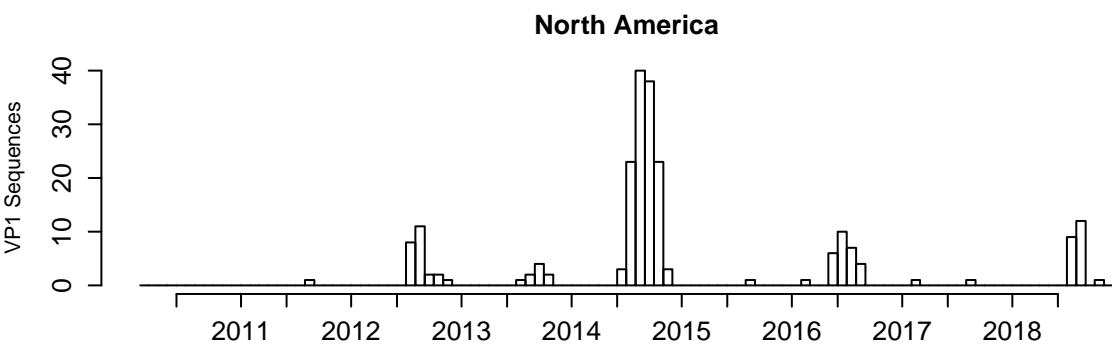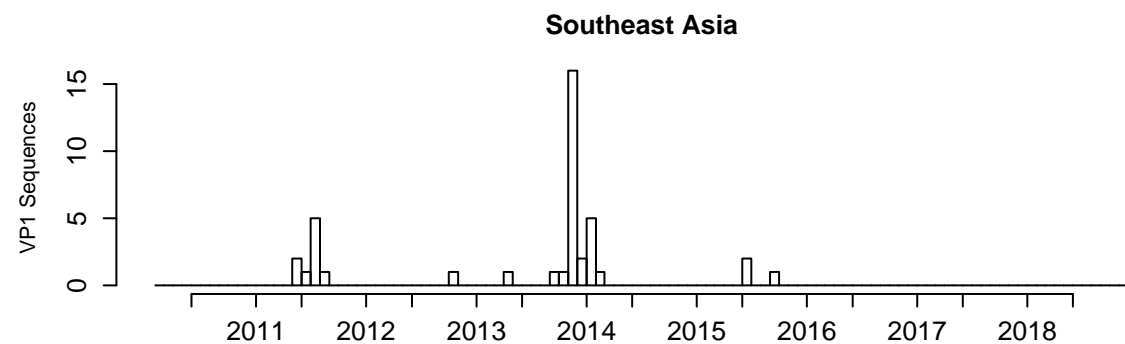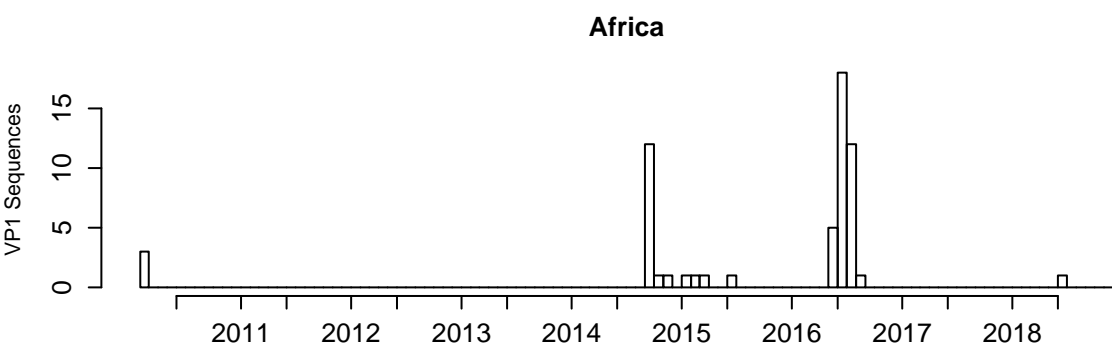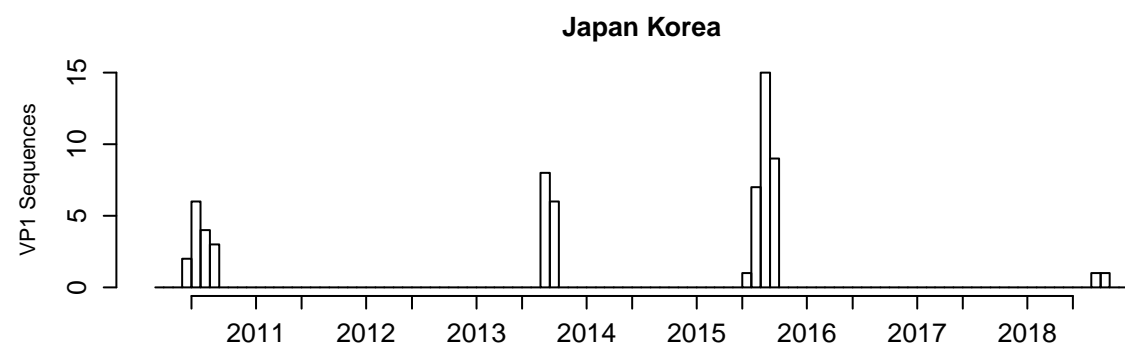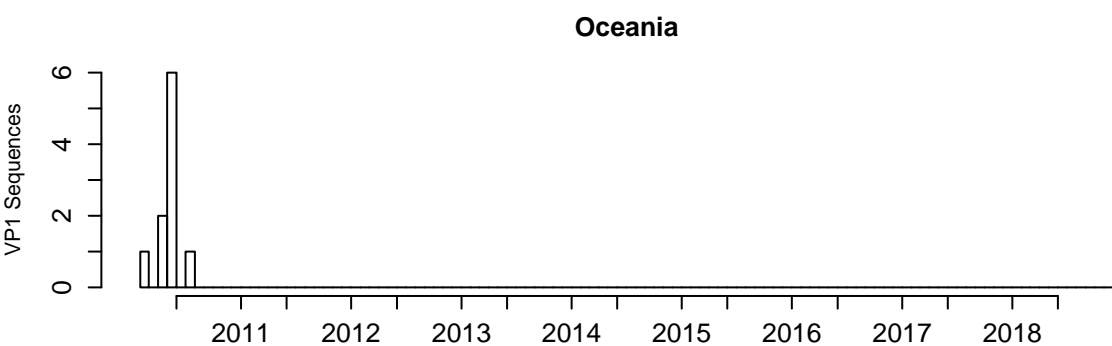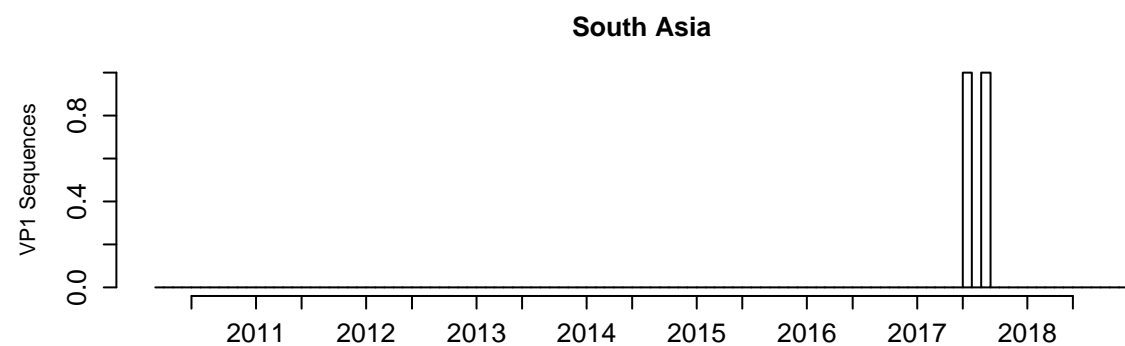

Supplement: S7 Fig — The number of VP1 sequences per month from 2010 until the end of 2018 is shown per region. The biennial autumn outbreak pattern in Europe and North America is apparent. The lack of sequences in many regions makes patterns hard to discern. (PDF) [file ppat.1010515.s008.pdf]

A)

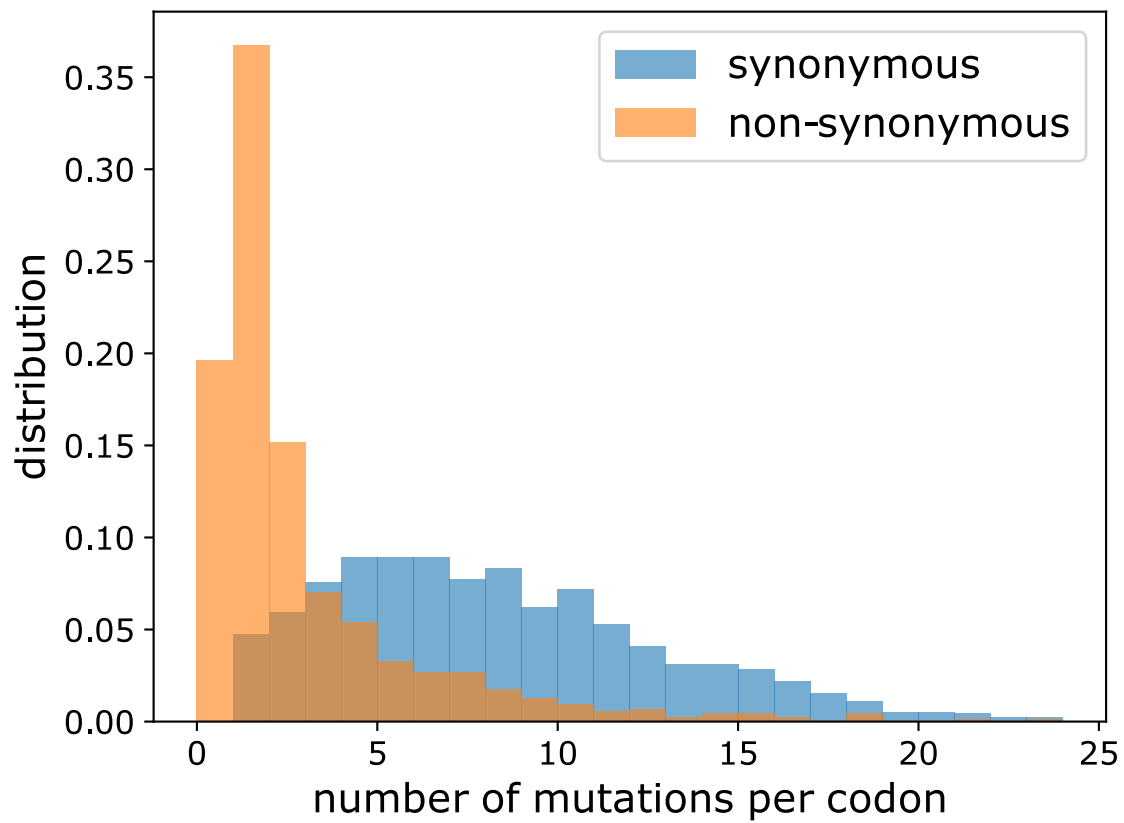

B)

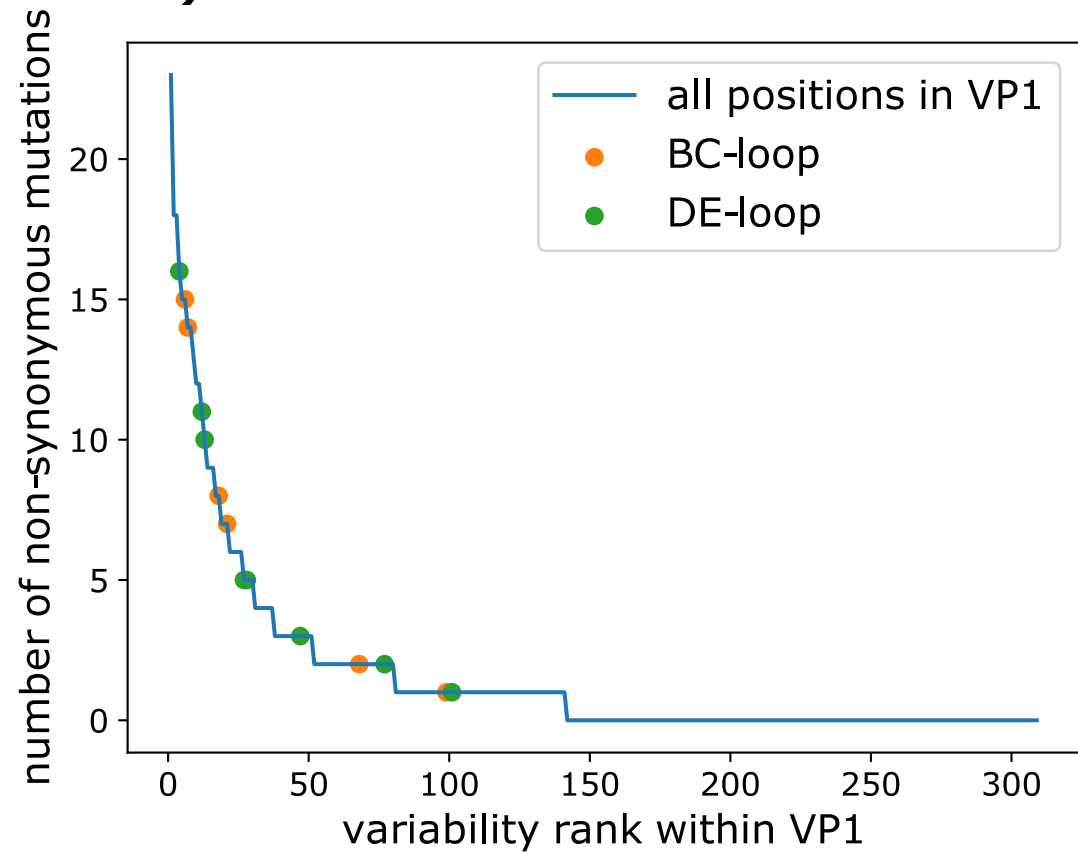

Supplement: S9 Fig — A) The distribution of synonymous and non-synonymous changes in VP1 shows that most sites have between 5–10 synonymous changes, but 0 or 1 non-synonymous change, suggesting most of protein is under purifying selection. However, some sites have over 15 non-synonymous changes, suggesting selection for diversification. B) When sites are ordered by their number of non-synonymous changes, codons within the BC- and DE-loops are among the most variable positions. (PDF) [file ppat.1010515.s010.pdf]
