# Supplementary material for: Evolution, geographic spreading, and demographic distribution of Enterovirus D68
Source: PLoS Pathog. 2022 May 31;18(5):e1010515. doi: 10.1371/journal.ppat.1010515 (PMC9212145; doi:10.1371/journal.ppat.1010515)
Supplement: S8 Fig — This figure extends from Fig 4D and 4E, but additionally includes the cumulative counts of amino-acid changes relative to the most recently reliable identifiable sequence (marked with ‘0’) along the branches. (PDF) [file ppat.1010515.s009.pdf]

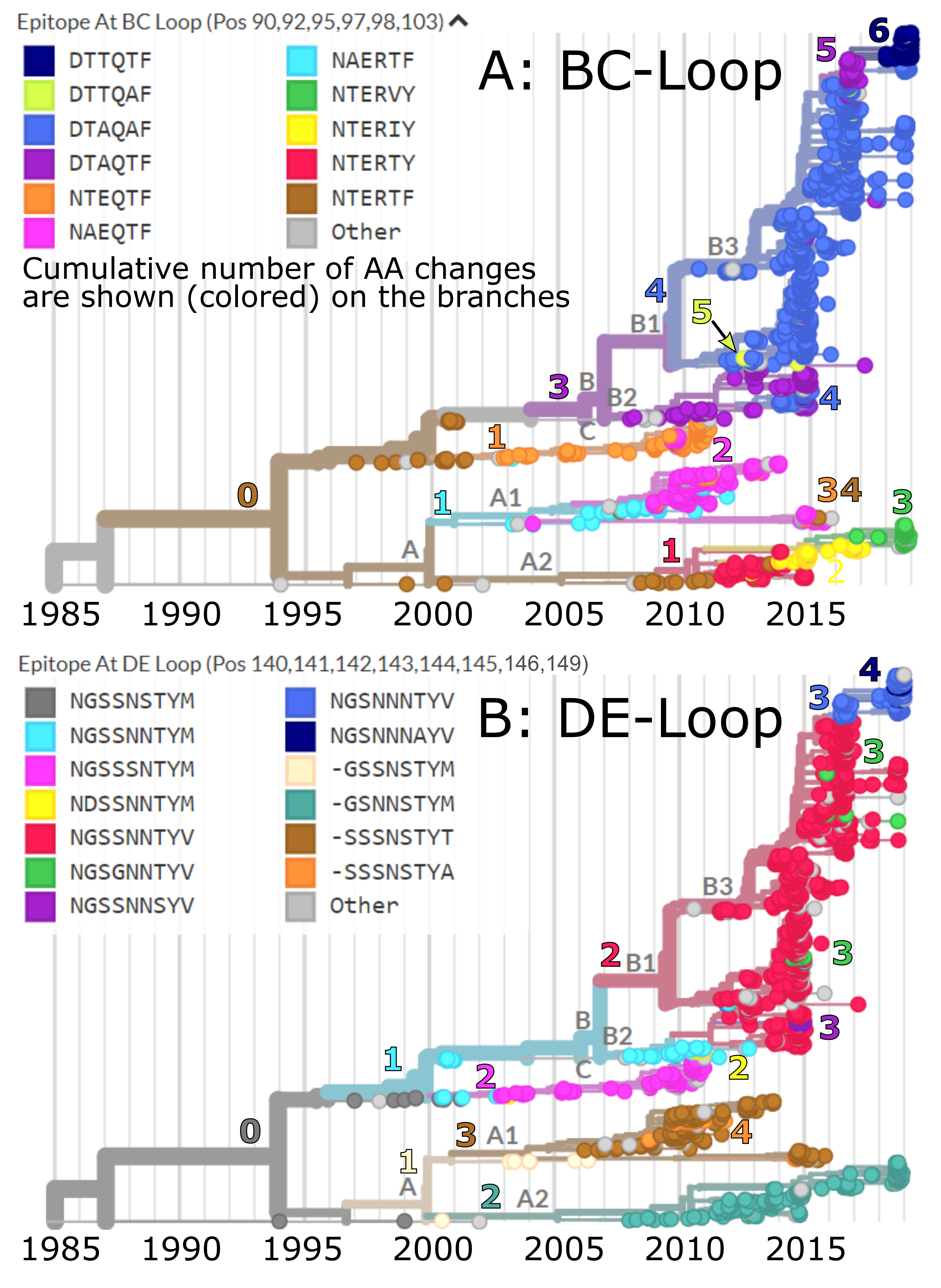
Epitope At BC Loop (Pos 90,92,95,97,98,103)
DTTQTF
DTTQAF
DTAQAF
DTAQTF
NTEQTF
NAEQTF
NAERTF
NTERVY
NTERIY
NTERTY
NTERTF
Other
A: BC-Loop
Cumulative number of AA changes are shown (colored) on the branches
0
1
A1
A
A2
1
1
2
3
B
B2
C
B1
B3
4
5
5
6
4
34
3
2
1985
1990
1995
2000
2005
2010
2015
Epitope At DE Loop (Pos 140,141,142,143,144,145,146,149)
NGSSNSTYM
NGSSNNTYM
NGSSSNTYM
NDSSNNTYM
NGSSNNTYV
NGSGNNTYV
NGSSNNSYV
NGSNNNTYV
NGSNNNAYV
-GSSNSTYM
-GSNNSTYM
-SSSNSTYT
-SSSNSTYA
Other
B: DE-Loop
0
1
2
C
B
B2
2
B1
B3
3
3
3
3
3
4
2
1
A
3
A1
4
2
A2
1985
1990
1995
2000
2005
2010
2015
