# Supplementary material for: Evolution, geographic spreading, and demographic distribution of Enterovirus D68
Source: PLoS Pathog. 2022 May 31;18(5):e1010515. doi: 10.1371/journal.ppat.1010515 (PMC9212145; doi:10.1371/journal.ppat.1010515)
Supplement: Attachment — Submitted filename: responses_to_reviewers.pdf [file ppat.1010515.s019.pdf]

RESPONSE TO REVIEWERS

Dear Editor

We thank Dr. Elde and Dr. Lauring for coordinating the review process and to the reviewers for providing these helpful and constructive reviews. We do our best below to address these comments and have modified the manuscript accordingly. A version with tracked changes is supplied along with a clean version.

REFEREE #1 (REMARKS TO THE AUTHOR)

R1 - 1: 1.1 In the introduction, I would have liked to read some more details about D68, e.g. is it an RNA virus, what is the typical course of infection, what are its transmission routes?
Response: *We thank the reviewer for this suggestion, and have added two sentences to the introduction describing the virus, its transmission, and clinical course of infection (lines 51-56).*

R1 - 2: 1.2 More on the epidemiological side, it would be interesting to have an idea of variations in incidence on a geographical and temporal scale (e.g. by showing weekly or monthly reports per region of the world). It would also be good to have an idea of the yearly incidence (or of the unknowns regarding this incidence).
Response: *We agree with the reviewer that the patterns of EV-D68 outbreaks are interesting and showing this more clearly would be ideal. Unfortunately, case data for EV-D68 is not readily available for most countries, and compiling it is beyond the scope of this paper. However, to give an idea of the patterns observed, we have added Fig. S 7, which shows the number of EV-D68 VP1 sequences over time, by region, and reference this in the introduction.*

R1 - 3: 1.3 I was wondering whether other studies have used sequence datasets of comparable size to study the worldwide genetic structure and epidemiology of the virus? Because currently, it is difficult to assess the originality of the study with that respect. Furthermore, I was wondering why the analyses focus in particular on 2014, 2016, and 2018 when there seem to be several samples collected before that date.
Response: *As far as we are aware, our study is unique in attempting to use all publicly available sequences to assess worldwide genetic structure and epidemiology over a longer time period. To our knowledge, this is also the first study to sample and whole-genome sequence EV-D68 across several European countries. Previous studies that have looked at genetic outbreaks have focused on specific countries or on one outbreak season, which is why we believe taking a wider and longer-term view is a valuable contribution to the field. We chose to focus on the 2014, 2016, and 2018 outbreaks as this is when EV-D68 surveillance began or became more systematic in many countries, due to to the association with AFM during the 2014 outbreak. Prior to this, we are less confident that the collected sequences represent wider circulation; for example, in 2012 of the 96 VP1 samples available, 44 are from Europe, and 32 come from France.*

R1 - 4: 2. As in any epidemiological study, a key issue is sampling. Overall, I think the authors could perhaps improve the demonstration that their sampling is exhaustive.
Response: *As the reviewer notes, sampling coverage is critical for this kind of epidemiological analysis. Our sampling is not exhaustive, but we were pleased to be able to cover multiple diverse geographies in Europe, including Stockholm, Sweden; Groningen, the Netherlands; Leuven, Belgium; Barcelona, Spain; Basel, Switzerland; and Tenerife, Canary Islands, Spain in our collection during 2018, and to include all good-quality publicly available sequences from around the world. All of our samples in 2018 came from specific clinics or hospitals, so will miss cases where the patient was taken to different healthcare centers or was not taken to see healthcare specialists. As most EV-D68 infections are mild, most cases likely will not seek care or be sampled. However, samples from different ages, countries, and sources are mixed on our tree, suggesting that we are capturing the circulating diversity.*

R1 - 5: 2.1 The meta-data associated with the 53 original genomes generated in the study is very detailed but for

the other genomes and the VP1 sequences the information is limited. Adding a summary table for all of the data would be great to rapidly visualise the coverage per region of the world and per year.

Response: *We thank the reviewer for the suggestion of an additional table to clarify the sequence coverage over time. We have added two supplementary tables, Table S VII and Table S VIII, which show the total number of VP1 and whole-genome sequences, respectively, per region per year. We have referenced these tables in lines 119-120.*

R1 - 6: 2.2 The temporal coverage seems very variable. For instance, based on Figure 2C, there were 2.5 more sequences collected in 2014 than in 2016 or 2018. Is this related to differences in the magnitude of the yearly epidemics? Could this also explain the lower diversity, as, for instance, in Figure 3B?

Response: *The 2014 outbreak included a large number of AFM cases, particularly in the US, which increased attention to the issue, this may have led to more sampling in this outbreak compared to those in 2016 and 2018. However, predictions based on sample screening data suggest the 2014 outbreak was genuinely larger (Park* et al., *2021)).*

From sequences, we know that the 2014 outbreak was also very diverse, when we look at the subclades circulating: B3, B2, B1, A1 and A2. In 2016 and 2018, the only subclades circulating were B3 and A2. Given that B1, B2, and A1 were not sampled in either outbreak (or since), we believe they have not been contributing meaningfully to circulation, rather than being under-sampled due to the lower level of sampling.

R1 - 7: 2.3 In several occurrences, the authors claim that the sampling is very dense in a geographical region but I think that the balance of this worldwide coverage could be better shown using the data.

Response: *We agree with the reviewer that providing the data makes it easier to see the sampling density difference between different regions. As mentioned in the answer to the question 2.1, we have now added two supplementary tables, Table S VII and Table S VIII, which show the total number of VP1 and whole-genome sequences, respectively, per region per year. We have referenced these tables in lines 119-120.*

R1 - 8: 2.4 The authors favour a (nice) hypothesis revolving around age-based sensitivity to virus lineages but could the uneven age distribution between the sampling years (2014 vs. 2016 and 2018) be caused by a sampling bias? (see also comment 3.4 below)

Response: *The reviewer is right in pointing out that sampling biases in ages could be problematic. It is true that due to the high number of AFM cases observed in the 2014 outbreak, this may have led to more children being tested (as AFM is overwhelmingly observed in children). However, our hypothesis around age-sensitivity is focused more on the relationship between subclade and age, rather than general age distribution. When sampling, the subclade of EV-D68 is unknown (indeed, in a clinical setting enteroviruses in respiratory specimens are generally detected by use of multiplex panels of respiratory pathogens and so the nature of the causative virus itself is unknown at sampling) so we expect sampling to reflect what is circulating in each age group. Though we see the A clade over-represented in older age groups, it is still primarily sampled in children (Fig 3), along with all the clades/sub-clades, meaning that increased sampling in children in 2014 would not be expected to bias our observations. Further, as EV-D68 very rarely causes AFM in adults, adults are unlikely to be screened for AFM, and are most likely being diagnosed with EV-D68 as part of routine respiratory virus screening during all three outbreaks, making it unlikely to bias sampling over time. Finally, the increase in sampling of adults more recently may be due to the A1 and A2 sublineages causing more symptomatic disease in adults, leading to more diagnoses, but this is difficult to show with the data we have.*

R1 - 9: 3.1 I was unsure I grasped the message about the (very nice) plot in Figure 2C between lineage coalescence and sampling. It seems intuitive that a peak in sampling would generate a peak in coalescence right before. However, perhaps the relative width of the coalescence peak with respect to the sampling peak could be related to the genetic diversity in the samples?

Response: *We thank the reviewer for pointing out that our explanation of Fig 2C could benefit from clarification. The reviewer is correct in their understanding of the plot - while it's intuitive that the peak of coalescence is prior to the sampling peak, if the majority of diversity was generated immediately prior to the outbreak, we would expect very high, narrow peaks, rather than the broader peaks we observe, which suggest substantial circulation prior to outbreak*

periods. We have clarified our explanation for this figure in the text in lines 170-172.

R1 - 10: 3.2 My expertise in epitope evolution is very limited but naively I would think that the clustering should be very strong if the authors show amino acid substitutions on a phylogeny. Wouldn't it be possible to have a more phenotypic measure as a label for the tree? That being said, apparently, the DTAQTF seems to revert in the BC-loop, which could be interesting but it is not discussed.

Response: *The reviewer is absolutely correct in pointing out that it is not surprising to see clustering of the epitope patterns on the tree. However, what we aim to convey in Fig 4 is not the clustering, but instead the pattern of change over time, which supports the hypothesis these regions are evolving antigenically. The trees on which these epitope patterns are shown are made with the whole VP1 region, so are supported by more than just the changes displayed. As the reviewer points out, the deviation away from clustering is also what's of interest here, as with the 'DTAQTF' pattern that appears twice separately due to the later reversion, which may suggest an advantage to this pattern. However, though interesting, as we only observe this a couple of times in the different epitopes, we refrained from commenting on these homoplasic patterns.*

To display the number of changes more clearly, we have added the number of amino-acid mutations from the most recently reliably identifiable epitope sequence (marked with '0') along each branch, in a zoomed version of the D & E panels from Fig 4 (to improve visibility) in new Fig. S 8 and also to Fig. S 6.

R1 - 11: 3.3 Still about the epitope evolution, I think some statistical support would be good for the statements regarding the fact that variability is mostly found on the outer surface of the protein.

Response: *In VP1 to VP4, there are 6 times as many mutations per site at residues on the outer surface of the capsid than at residues that are either buried or in the interior of the capsid. A Fisher exact test yields an odds ratio of 6 and a p-value* $< 10^{-16}$*. The proteins VP1, VP2, and VP3 are also individually significantly enriched for mutations on the outer surface with the weakest signal in VP3 (OR=2.7, p-value 0.0032). We have added these results to the text, lines 253-256, and in the supplementary methods, lines 706-710.*

R1 - 12: 3.4 As an evolutionary biologist interested in infection virulence, I was very enthusiastic about the correlation found between virus lineage and patient age. However, I have two comments. First, from a statistical point of view, I was surprised to see the authors use a linear model to study the association because it de facto assumes that the lineages are independent. In other words, this is a clear instance where correcting for phylogenetic non-independence is important. Second, Figure 3B suggests that there is an uneven sampling in terms of age between the years. If the authors could show that this association with age is independent of the sampling year, either by adding it as a cofactor in the model or by only using the 2014 year, I think that would strengthen their result.

Response: *We thank the reviewer for these two insightful comments. In the first comment, the reviewer is astute in noting that the phylogenetic relationship is important. However, here by looking at the subclades we focus on looking at fairly deep splits in the tree that have existed for several outbreak years, rather than closer phylogenetic relationships near the tips. Within the larger splits, we are indeed looking to see if those groups (the subclades) are associated with differences in age which we propose are indeed a phylogenetic trait.*

In the second comment, we agree with the reviewer that adding year as a co-factor to the linear model is of interest to ensure there is no impact due to differences in different sampling periods. When year was added, the association with higher age for subclades A1 and A2 remained significant (p<0.001 in both cases). We also added region as a co-factor, as sampling practices may differ from region to region, and again the association remained significant ($p < 0.00006$ *in both cases). We have added this to the text on lines 229-231 and in the supplementary methods on line 690.*

R1 - 13: 4.1 The authors lean on a preprint from wastewater samples. In addition to the legitimate question about the validity of this work, the method used is itself very experimental. I could be wrong but I expect wastewater samples to be very fragmented and I am unsure about the confidence we can have regarding the genetic composition of the sequences retrieved.

Response: *The reviewer is correct to consider the quality of wastewater samples, which can indeed be fragmented*

and inappropriate to include in such analyses. In particular, having dealt with wastewater samples during the SARS-CoV-2 pandemic, we are aware of how their fragmentation, incompleteness, and tendency to be mixtures of many variants can impact phylogenies - though this also causes them to stand out quite noticeably. However, the included sequences (from Majumdar et al. [42]) were Sanger sequenced and are approximately 850bp in length, which increases the likelihood that these are from individual viruses and that fragmentation is not likely not an issue. To highlight this and assuage concern, we have added that these were Sanger sequenced to line 303.

Further, in updating the EV-D68 VP1 build post-pandemic, we have received sewage ('SWG') samples from the UK from late 2018 and 2021 (also Sanger sequenced), which also seem of very good quality and again intermix with clinical swab samples (this work is not complete and not yet fully publicly available, but can be seen to some extent in a draft build at this link). Finally, apart the conclusions of the preprint from which these VP1 wastewater samples come, in our manuscript we are looking only at how these samples mix with others in our analysis.

R1 - 14: 4.2 The paragraph about serology reads also strangely. The authors describe in detail an earlier study by Harrison et al (2019, Emerg Infect Dis) to discuss sera from individuals react differently against viruses based on their ages to support the idea that individuals are reinfected. The problem is that two paragraphs are a bit short to prove this idea. Furthermore, the authors invoke the original antigenic sin (which would benefit from a reference) to explain that older individuals would be infected by lineage A2, but I do not understand why if so we shouldn't see infections in older individuals by other lineages (especially B3 that is more divergent). If this is the motivation of the study, I think it should be present from the introduction and the main hurdles should be explained clearly (i.e. the fact that "young" adults are asymptomatic and rarely sampled).

Response: *We thank the reviewer for pointing out our oversight in not including a reference to antigenic sin, and have included this now (line 378). The reviewer is correct in suggesting that the evidence for proof of antigenic sin being behind the difference in ages we observe in different clades is still incomplete at this point. While we feel this hypothesis is an important one to put forward, stemming from the results of our work in this paper, it is indeed one that we are putting forth in our discussion and needs further directed study, rather than one we feel we can prove in this manuscript. We have tried to clarify that this is a hypothesis that needs further investigation by adding a sentence in lines 384-387.*

R1 - 15: 4.3 I found the mentioning of SARS-CoV-2 strange, partly because there was no scientific reference to back up the claims made, and also partly because of the lack of biological justification. If the authors wish to draw a parallel between D68 and SARS-CoV-2 I think they should make a better case as to how the two are related. Intuitively, I would have said that a reference to seasonal coronaviruses would have been more appropriate here

Response: *Upon rereading this section, we agree that the reason for bringing up SARS-CoV-2 could be made clearer. Our intention was to highlight how the current situation has cast into a sharp light how much we still have to learn about viral immunity, evolution, and transmission - this has been particularly key with concerns about new SARS-CoV-2 variants and vaccine efficacy over time. Though EV-D68 is not closely related to SARS-CoV-2, we feel that a better understanding of these properties in all viruses will be the only way to build a solid base that will help us be prepared for novel ones. We have tried to make this clearer by editing the end of the paper and removing the sentence specifically referencing the outcome differences by age observed for SARS-CoV-2 (lines 396-400).*

Minor issues

R1 - 16: abstract: I am unsure about the formulation of the first sentence. Can we really say that an outbreak of enterovirus cased a disease? Intuitively, I would say that a virus caused an outbreak of disease or that a virus outbreak caused an increase in diseases.

Response: *We thank the reviewer for pointing out this imperfect wording; we have adjusted the opening sentence to follow the reviewer's suggestion.*

l.47: Add a reference for the 2014 outbreaks?

Response: *We thank the reviewer for spotting this oversight, and have added a reference to the 2014 outbreaks to the first sentence of the introduction.*

Figure 1: How were the "key" clusters defined?

Response: *We have clarified in the legend for Figure 1 that the 'key' clusters from 2018 were the largest in the outbreak.*

l.130: Please further explain why the epidemic was "well-sampled" in 2015-2016.

Response: *We thank the reviewer for pointing out that this was not clear, and have added that the B3 subclade was relatively well sampled in 2015-2016, having more sequences during this period than any other subclade in any other 2-year period.*

l.132-133: I was unable to determine whether this sentence was referring to one of the figures.

Response: *This sentence was indeed unclear; we have removed 'and vice versa' as this is not clearly supported and added a reference to the overview of Fig 1 A, and to the online view (as this is hard to see in the embedded figure).*

l.133-134: Same comment here: can this be back-up by one of the figures?

Response: *We have clarified this sentence and added a reference to Figure 1 B, zoomed cluster III.*

l.137-138: Does this correspond to Figure 1B?

Response: *We have added a reference to Figure 1, where the MRCAs of the subclades can be seen (in both the overview and zoomed views), to the first line of this section.*

l.141-148: Knowing that the lineage originates in early 2017, it is probably feasible to estimate how much secondary infections this initial "common ancestor" would have generated early 2018. I think it would help to determine whether it would be feasible to have this lineage represented (here the authors do have 3 full genomes from early 2018).

Response: *We agree with the reviewer that having a better understanding of how viruses circulating in non-outbreak periods contribute to onward transmissions, and ultimately outbreaks, would be very insightful. Unfortunately, we know very little about how and how much EV-D68 transmits during non-outbreak periods, or how the virus moves between the observed geographic regions, so this is not something we are able to estimate. Phylodynamic estimates of population size/coalescence rate suggest a rapid increase in the second quarter of 2018 (Supp Fig 3).*

l.169: Here it would be interesting to know more about the virus circulation. Is it like influenza where Asia acts as a reservoir? Is the virus seasonal in Europe and the USA? In this case, "underreporting" in a country could also be due to the fact that there are no cases in this country.

Response: *We agree with the reviewer that it would be incredibly interesting to know more about the seasonal and global circulation of EV-D68. While it is clear that there is large-scale circulation across the world, with most children infected before they turn 10, relatively little is currently known about dynamics and persistence, which is one of the drivers both for the current manuscript and for calling for future work. EV-D68 is strongly seasonal and biennial in the USA and Europe, as we note in our introduction, and seems to be similarly seasonal in other countries, though there is less long-term data available. While it's possible that EV-D68 could have a reservoir in Asia or elsewhere, currently there is little data to support this hypothesis - European/North American clusters sometimes, but not always, have ancestors in Asia. However, since the available data suggests EV-D68 is seasonal worldwide, this would still suggest under-reporting of cases between seasons and/or in countries where it may not be seasonal.*

l.195: It would be good to provide quantitative support for the claim that the continents were "thoroughly sampled".

Response: *We have edited this line to say 'relatively well-sampled' rather than 'thoroughly,' and reference supplementary Table S VII.*

l.300: Titers refers to antibody titers correct?

Response: *We thank the reviewer for spotting this oversight and have added 'antibody' to this sentence to clarify.*

l.305: A high substitution rate compared to what?

Response: *We have clarified this sentence by noting that the rate is high compared the rest of the genome but similar to that of DE and BC loop epitopes, which are known to evolve more quickly.*

l.345: I think a reference is needed for the original antigenic sin

Response: *We have now included a reference for original antigenic sin, as noted in our response* ***R1- 14*** */ 4.2.*

Some of the figures were difficult to interpret. For instance, in Figure 2B and 2C it is unclear what lineages are considered (are these all the lineages shown in Figure 1A?). Speaking of Figure 1A, it would actually help to label the panels so that they can be mentioned in the text.

Response: *As suggested by the reviewer, we have added panel labels to Figure 1 and referenced these in the text, and in response to some of the other queries above. We thank the reviewer for letting us know that the construction of Figure 2B & C was not clear. We have clarified in the supplementary methods (section F) and in the Figure 2 legend that all tips sampled during each outbreak year are included. We have also clarified the Figure 2 legend to say that the lineages are simply the common ancestors of all samples in an outbreak year, which should help differentiate them from the clusters shown in Figure 1.*

l.645: What was the substitution model used for the phylogeny inference?

Response: *A GTR substitution model was used for phylogeny inference, and this was clarified in the supplementary methods, section C.*

Suppl Table IV: What does `?10^3` mean in the coverage column?

Response: *We thank the reviewer for spotting this formatting error - this should be* $\geq 10^3$ *and has been corrected. The meaning of these coverage values is explained in text at the bottom of the file.*

REFEREE #2 (REMARKS TO THE AUTHOR)

R2 - 1: A key conclusion here is that D68 is apparently under continuous antigenic evolution, a not unexpected finding or a novel one. To predict which pathogen may lead to the next viral pandemic requires a very different set of judgements that are not answered here. Pandemics require one (or more) novel, transmissible serotypes to be introduced into an immunologically naive population.

Response: *We agree with the reviewer that studies previous to ours have identified signals of selection pressure in the epitope regions of the VP1 gene of EV-D68, as we cite in the third paragraph of our introduction. However, we believe our study provides more evidence that this change is indeed antigenic evolution by looking at the changes in epitope sequences and their location on the viral capsid.*

We also agree with the reviewer that predicting the next pathogen that may cause a pandemic is very difficult, which is why we wrote, "It is impossible to predict which pathogen may lead to the next viral pandemic," and then continue this sentence highlighting the importance of better understanding viral dynamics, evolution, and immunity in order to be prepared for future pandemic pathogens (last paragraph of discussion).

R2 - 2: 1. EV-D68 is a defined EV genotype, meaning isolates are binned here by sequencing similarity thresholds not (as in old days), serotyping. Certainly sequence clades A, B, C are under positive selection as measured by diversity in certain loop elements, but is there any evidence that fresh convalescent human serum from any recently infected individual (e.g. clade A), will not cross-neutralize viruses from the other clades? Doesnt have to be the same high titer, but will it cross-neutralize at even a partially protective titer (perhaps 1:5)?

Response: *EV-D68 is a serotype as well as a genotype, so we do expect some cross-neutralization between the different subclades, as shown by Harrison et al. (Emerg Inf Dis 2019). The reviewer is also correct in asserting that titers levels alone do not fully predict how effective protection against future infection may be, though they are highly correlated in SARS-CoV-2 (for example, Khoury* et al. *(2021)). Unfortunately, more in-depth studies on antibody titers for EV-D68 are not available, and more work in this area would surely help us better understand both the cross-reactivity between subclades and what protection this confers.*

R2 - 3: 2. If "no" then all presented data really just support the idea that D68 as a serotype is not evolving linearly, but may just continually recycle some immunogenic, permissible loop configurations, albeit many decades apart. Flu does this, for example, and leaves the same type of host age-dependent patterns as the clades rise, fall,

and eventually recycle. By definition, this evolution pattern may lead to continuous outbreaks in temporarily naive geographic regions, but not pandemics

Response: *We agree with the reviewer that it is possible that EV-D68 may be recycling epitope configurations over time, and this would be an interesting hypothesis to explore further. Unfortunately, however, the lack of sequences over very long time periods limit our ability to investigate whether this may be true. Hopefully, as more sequences are gathered over the coming years, and as more serological studies are carried out, this hypothesis can be further explored.*

R2 - 4: 3. If yes then are the data telling us that D-68 is really on the verge of becoming D-69, or D-98 or something else that is so immunologically distinct and different, it has the potential for a novel pandemic?

Response: *The reviewer raises an interesting possibility, but we do not believe that the current data supports an imminent serological change or pandemic potential. Currently an enterovirus species is defined as having >70% nucleotide or >88% amino acid sequence similarity (Oberste* et al.*, 1999). Currently, sequence similarity for the VP1 sequences we include here, relative to our 2016 reference sequence (*`KX675261`*), is >81% similar by nucleotide sequence. However, even without complete loss of neutralization or becoming a new serotype, changes in immunity due to antigenic evolution are still informative and could be clinically relevant.*

R2 - 5: 4. Are there known structural constraints on the capsid or receptor binding that might differentiate between yes and no?

Response: *There are some indications of structural constraints, for example, residues of the floor of the canyon which harbours the receptor binding site has conserved motifs in VP1 and VP3 across the picornaviruses (Rossmann* et al.*, 1985). We see the same pattern of less variation in the 'canyon residues' in EV-D68 (amino acids VP1 codons 146-155 and VP3 codons 132-141), except for codon 148 in VP1. On the other hand, the higher evolutionary rate in the loops likely indicates these are less constrained. We have added a line on the lesser variation of 'canyon' sites on lines 256-258.*

R2 - 6: 5. I might have missed it, but do the Nim2 and Nim3 epitope sequences (check the sequence analogs in polio or the RVs) show similar patterns of clade-specific variability?

Response: *Our efforts to identify the Nim2 and Nim3 epitope regions in EV-D68 suggest that Nim-II is sites 225-242 in VP1 and 161-169 in VP2. At these position we found 3 sequences, 2 with different bases at VP1:227 and 1 with a different base at VP2:167, but nothing associated with the subclades.*

Nim-III is is 72-79 and 208 in VP3. Here, we see a change in VP3:73 from V to A where the B and C clade splits off in the phylogeny. Further, we see a change in VP3:208 from D to N where the A clades split off from the phylogeny.

While we do see these 2 positions in Nim-III with deep clade variability, it seems the BC and DE loops and C-terminus may be more important locations for epitope variability in EV-D68.

REFEREE #3 (REMARKS TO THE AUTHOR)

R3 - 1: In the introduction, the authors write: .. the degree to which EV-D68 evolution is driven by immune escape or whether incidence patterns are determined by pre-existing immunity is unclear. I could not see much distinction between these two, perhaps rephrase.

Response: *We thank the reviewer for pointing this out, and have reworded this sentence.*

R3 - 2: 2. The analysis of antigenic evolution (pages 11 onward) is very descriptive and less quantitative. For example, I would have expected a site-specific dn/ds analysis here that can quantify positive selection or even contrast between rate of evolution of different sites. Also, it would have been nice to see a quantitative measure of the imbalance of the phylogeny, which once again supports the notion of antigenic drift.

Response: *We appreciate the suggestion from the reviewer to add some quantitative analyses to the antigenic*

evolution section. For the BC and DE loops, standard dN/dS analyses largely come up non-significant for positive selection, because of the uniformly high number and near saturation of synonymous mutations. Specific positions in the BC and DE loops are, however, among the sites with highest number of non-synonymous mutations. To further quantify evolution in VP1, and these loops in particular, we have added an additional figure, Fig. S 9. In comparing the distribution of synonymous and non-synonymous changes, most sites have 5-10 synonymous changes and 0 or 1 non-synonymous changes showing purifying selection in most parts of VP1. But some codons have over 15 non-synonymous changes. Looking at where the codons in the BC- and DE-loops lie within the distribution of the non-synonymous changes, we can see they are among the most variable positions. This is added in the text on lines 259-263.

Additionally, we have added a new statistical comparison of the number of changes in sites that are exposed on the capsid versus those that are buried or internal – please see the response to reviewer comment R1-11.

R3 - 3: 3. Would be nice to add to panels D and E of figure 4, how many amino-acid replacements are there between the different color coded epitope?

Response: *We thank the reviewer for this suggestion, and have added a zoomed version of the D & E panels of Fig 4 as new Fig. S 8. This has the cumulative number of amino-acid changes on the branches, color-coded to make association easier. Additionally, we have added the same counts to Fig. S 6 (the phylogeny colored by the C-terminal AA sequence).*

R3 - 4: 4. The authors discuss how antigenic evolution drives diversification of the virus, and also mention the biennial pattern of infection of this virus. Antigenic evolution has been described for many viruses (influenza, seasonal coronaviruses). Could the authors discuss and raise hypotheses why there is a biennial pattern (rather than annual) pattern and if and how this is related to re-infections of adults?

Response: *A study that modelled the dynamics of non-polio enteroviruses in Japan showed that transmission dynamics were driven by serotype-specific immunity, and circulation patterns were determined by birth rate (Pons-Salort and Grassly, 2018). The main model assumed a life-long immunity after infection but birth rate remained the determining factor also when extending the model with waning immunity. Similarly, modelling based on the predictions of EV-D68 cases detected in routine diagnostics in the US showed the particular combination of EV-D68's seasonal forcing and basic reproductive number to generate a biennial pattern (and also not in all states) and that this biennial pattern could be a transient feature (also this SIR model assumed life-long immunity after infection) (Park* et al.*, 2021). Thus the biennial pattern seems to be caused by the time it takes for a sufficiently large susceptible cohort to emerge. As discussed in the manuscript, a high degree of geographical intermixing of sequences can indicate seeding by adults having asymptomatic reinfections. We have added two sentences discussing the build-up of a naive population and the potentially transient nature of the pattern to the introduction (lines 61-65).*

Finally, even in influenza and seasonal coronaviruses, their patterns of circulation and outbreaks are not precisely annual, in that strains or lineages circulate at different frequencies in different years. In influenza, we typically see one or two of the four lineages in a season. In seasonal coronaviruses, we can see that within the Alpha- and Beta-coronaviruses, there is an alternating pattern, meaning that each virus has something like a biennial pattern (Dyrdak et al.*, 2021).*

REFERENCES

Dyrdak, R., E. B. Hodcroft, M. Wahlund, R. A. Neher, and J. Albert (2021), Journal of Clinical Virology: The Official Publication of the Pan American Society for Clinical Virology **136**, 104754.

Khoury, D. S., D. Cromer, A. Reynaldi, T. E. Schlub, A. K. Wheatley, J. A. Juno, K. Subbarao, S. J. Kent, J. A. Triccas, and M. P. Davenport (2021), Nature Medicine **27** (7), 1205, number: 7 Publisher: Nature Publishing Group.

Oberste, M. S., K. Maher, D. R. Kilpatrick, and M. A. Pallansch (1999), Journal of virology **73** (3), 1941.

Park, S. W., M. Pons-Salort, K. Messacar, C. Cook, L. Meyers, J. Farrar, and B. T. Grenfell (2021), Science Translational Medicine **13** (584).

Pons-Salort, M., and N. C. Grassly (2018), Science **361** (6404), 800.

Rossmann, M. G., E. Arnold, J. W. Erickson, E. A. Frankenberger, J. P. Griffith, H.-J. Hecht, J. E. Johnson, G. Kamer, M. Luo, A. G. Mosser, *et al.* (1985), Nature **317** (6033), 145.
